# A Bibliometric Analysis of Aging in COVID-19

**DOI:** 10.14336/AD.2022.0620

**Published:** 2023-02-01

**Authors:** Weiming Guo, Jinglei Zang, Jingfen Lu, Yanqiuzi Ma, Gang Fan

**Affiliations:** ^1^Huazhong University of Science and Technology Union Shenzhen Hospital, the 6^th^ Affiliated Hospital of Shenzhen University Health Science Center, Shenzhen 518000, China.; ^2^Changsha Health Vocational College, Changsha 410600, China.; ^3^Hunan University of Traditional Chinese Medicine, Changsha 410218, China.; ^4^The National Center for Chronic and Noncommunicable Disease Control and Prevention, Beijing 102206, China.

Dear Editor

The coronavirus (COVID-19) is raging worldwide, depriving hundreds of millions of people of the ability to live their normal lives and posing a major security threat to all humankind [1, 2]. As of May 24, 2022, 523,786,368 confirmed cases of 2019 coronavirus diseases, including 6,279,667 deaths, have been reported to the World Health Organization (WHO, https://covid19.who.int/). Aging causes numerous biological changes that result in age-related illnesses and susceptibility to infectious diseases [3, 4]. The COVID-19 pandemic has had a significant impact on older populations, aging is considered the most significant risk factor for severe COVID-19 disease and its adverse health outcomes [5]. We here present the age-related research on COVID-19 using a bibliometric analysis to illustrate the research landscape and explore the hot topics and emerging trends from a global perspective.

All data were retrieved from the Web of Science Core Collection (WoSCC, www.webofscience.com/wos/ woscc/basic-search) including SCI-EXPANDED by using the following search strategy: (TS = (“2019 Novel Coronavirus Disease” or “COVID-19" or "coronavirus 2” or “SARS-CoV-2” or “Novel coronavirus pneumonia” or “Novel Coronavirus" or "2019-nCoV” or “coronavirus disease 2019” or “coronavirus disease-19”)) and (TS = ("Aging" or “Biological Aging" or "Senescence" or "Aging, Biological" )) [6, 7]. Since the first article on aging and COVID-19 was published in 2020, the time range of data retrieval was set from January 1, 2020, to April 30, 2022. We used VOSviewer (version 1.6.18.0) and R Bibliometric (version 4.2.0) software to analyze the retrieval results about aging and COVID-19, including the distribution of countries, cumulative publications, collaborations, thematic evolution keywords, and WoS categories.

A total of 1,282 publications, including 1,049 articles and 322 reviews, were extracted from the WoS database. The time curve of cumulative publications each year was constructed by the logistic regression models: y (2020) = 26.885x-36.167, R² =0.9332; y (2021) = 58.301x+46.212, R² = 0.998; and y (2022) = 26.885x-36.167, R² = 0.933. The distribution of relevant publications by month shows an increasing trend and the number of documents published in 2021 exhibits the strongest trend (Fig. 1A). As of the end of April 2022, 206 articles on aging and COVID-19 have been published, and the cumulative number of publications will be over 500 by the end of 2022.

Global contributions were analyzed according to the author’s nationality and represented by R Bibliometric in a world map (Fig. 1B). Eighty countries and regions were involved in the research on aging and COVID-19 and the articles were mainly published in North America, Asia, and European countries. The United States contributed the greatest number of articles (1,629, 30.2% of all scientific production), followed by China (472, 8.7%), and Canada (418, 7.7%) (Supplementary Table 1). The cross-country relationship map shows the collaboration between different countries on the research of aging and COVID-19 (Fig. 1C). The United States, Italy, Germany, Canada, and China were the top five countries that initiated a collaboration with other countries. The United States had the most foreign cooperation and had the closest cooperation with Canada (28 occurrences), China (25 occurrences), Germany (21 occurrences), and The United Kingdom (21 occurrences) (Supplementary Table 2).

**Figure 1.**
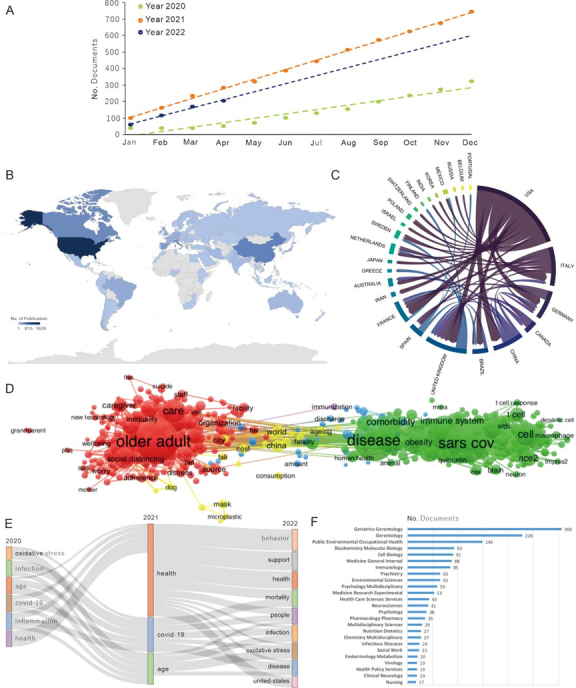
Worldwide research trends on aging inCOVID-19. (A) Monthly cumulative distribution of publications in the field ofaging and COVID-19. (B) Geographical distribution map of global publications regardingaging and COVID-19. (C) Distribution of publications in the field ofaging and COVID-19 according to the year. (D) Clustering co-occurrence map of the predominant keywords of studies onaging and COVID-19. (E) Thematic evolution from 2020 to 2022. F, the number of publications of categories.

Co-occurrence of keywords is beneficial for clarifying the main themes and establish a framework of research on aging and COVID-19. Here, 616 keywords with a co-occurrence >10 times were extracted from titles and abstracts (Supplementary Table 3) and were clustered into five categories represented by different colors (red, yellow, blue, and green) in Figure 1D. The top ten keywords included "older adult” (1,131 occurrences), "disease" (982 occurrences), "sarscov" (928 occurrences), "infection" (664 occurrences), "participant" (354 occurrences), "care" (311 occurrences), "cell" (300 occurrences), "loneliness" (267 occurrences), "mechanism" (239 occurrences), and “virus” (236 occurrences)”, among others.

The thematic evolution diagram was another important way to reflect the research hotspots, frontiers, and emerging trends over time (Fig. 1E). The top items, including “oxidative stress”, “infection”, “age”, “inflammation”, and “health”, suggest mainstream research in 2020. “Health” presents a prominent research issue in 2021. Most notably, the study of aging and COVID-19 in relation to “behavior”, “support”, and “health” have risen in 2022, indicating that these research directions have been getting attention in the “post-COVID-19” era and may also have the potential to become new research hotspots in the future.

The category analysis reflects research areas on aging and COVID-19, with the bar chart in Figure 1F showing the number of publications in the top 25 disciplines. The leading disciplines are “Geriatrics Gerontology”, which contains 306 articles (306 out of 1,420, accounting for 21.55% of the total), followed by “Gerontology” (228, 16.06%), and “Public Environmental Occupational Health” (149, 8.96%).

In this study, we assessed the scientific output and activity regarding aging and COVID-19 research from a global perspective via visual or cluster analysis, providing vivid and comprehensive information for other researchers. Some critical publications may have not been identified since only English articles from the WoSCC dataset were incorporated into the analysis. Despite this, the present study has included the vast majority of publications on aging and COVID-19 from 2020. Our results indicate that there will continue to be a dramatically increasing number of publications on aging and COVID-19 based on the current global trends. The United States, Canada, and China have made great contributions to the research field of aging and COVID-19. The United States and Italy are the two countries that have initiated the most cooperation in this field. The COVID-19 pandemic has created unprecedented challenges for older adults not only to physical health but also to mental health [8, 9]. Current research focuses on “behavior”, “support”, and “health”, indicating a proactive transformation from basic research to the research of mental health and social support to improve interventions and the quality of life of elderly patients with COVID-19 [10].

## Supplementary Materials

The Supplementary data can be found online at: www.aginganddisease.org/EN/10.14336/AD.2022.0620.
