# Supplementary material for: A Bibliometric Analysis of Aging in COVID-19
Source: Aging Dis. 2023 Feb 1;14(1):6–8. doi: 10.14336/AD.2022.0620 (PMC9937693; doi:10.14336/AD.2022.0620)
Supplement: Supplementary file 1 — The Supplementary data can be found online at: www.aginganddisease.org/EN/10.14336/AD.2022.0620. [file AD-14-1-6-s.pdf]

SUPPLEMENTARY DATA

A Bibliometric Analysis of Aging in COVID-19

Weiming Guo[1#], Jinglei Zang[2#], Jingfen Lu[3#], Yanqiuzi Ma[4], Gang Fan[1*]

[1]Huazhong University of Science and Technology Union Shenzhen Hospital, the 6th Affiliated Hospital of Shenzhen University Health Science Center, Shenzhen 518000, China. [2]Changsha Health Vocational College, Changsha 410600, China. [3]Hunan University of Traditional Chinese Medicine, Changsha 410218, China. [4]The National Center for Chronic and Noncommunicable Disease Control and Prevention, Beijing 102206, China.

© 2022. Guo W et al. Published online at http://www.aginganddisease.org/EN/10.14336/AD.2022.0620

SUPPLEMENTARY DATA

Supplementary Table 1. Total number of publications by countries.

Region	No.
USA	1629
CHINA	472
CANADA	418
ITALY	376
SPAIN	289
BRAZIL	193
GERMANY	174
UK	164
FRANCE	146
JAPAN	117
IRAN	108
AUSTRALIA	95
GREECE	79
INDIA	75
NETHERLANDS	75
ISRAEL	53
SWEDEN	53
SINGAPORE	51
SOUTH KOREA	51
MEXICO	49
FINLAND	43
ROMANIA	42
SWITZERLAND	40
POLAND	39
TURKEY	38
PORTUGAL	34
RUSSIA	33
MALAYSIA	32
AUSTRIA	31
HUNGARY	28
SAUDI ARABIA	23
SERBIA	20
BELGIUM	19
IRELAND	19
CHILE	17
DENMARK	17
EGYPT	16
ARGENTINA	14
NORWAY	14
CUBA	13
CZECH REPUBLIC	13
NIGERIA	12
ECUADOR	11
IRAQ	11
INDONESIA	10
QATAR	10
SLOVENIA	10
THAILAND	10
TUNISIA	10
CROATIA	8
BANGLADESH	6
LUXEMBOURG	6
NEW ZEALAND	6
PERU	6
SLOVAKIA	6
UKRAINE	6
COLOMBIA	5
PAKISTAN	5

© 2022. Guo W et al. Published online at http://www.aginganddisease.org/EN/10.14336/AD.2022.0620

ETHIOPIA	4
JORDAN	4
SOUTH AFRICA	4
SRI LANKA	4
LEBANON	3
LITHUANIA	3
PHILIPPINES	3
BAHRAIN	2
BARBADOS	2
CYPRUS	2
KENYA	2
LIECHTENSTEIN	2
MOLDOVA	2
BRUNEI	1
ESTONIA	1
KAZAKHSTAN	1
LATVIA	1
MALTA	1
MOROCCO	1
URUGUAY	1
VIETNAM	1
ZIMBABWE	1

Supplementary Table 2. Country Collaboration

From	To	occurrences
USA	CANADA	28
USA	CHINA	25
USA	GERMANY	21
USA	UNITED KINGDOM	21
USA	ITALY	20
USA	FRANCE	19
USA	BRAZIL	16
ITALY	UNITED KINGDOM	12
GERMANY	UNITED KINGDOM	11
USA	IRAN	11
CANADA	UNITED KINGDOM	10
ITALY	FRANCE	10
USA	AUSTRALIA	10
CHINA	AUSTRALIA	9
CHINA	GERMANY	9
BRAZIL	UNITED KINGDOM	8
CHINA	ITALY	8
CHINA	UNITED KINGDOM	8
UNITED KINGDOM	GREECE	8
USA	JAPAN	8
USA	NETHERLANDS	8
CANADA	GERMANY	7
CHINA	CANADA	7
GERMANY	FRANCE	7
ITALY	GERMANY	7
SPAIN	BRAZIL	7
UNITED KINGDOM	SWEDEN	7
USA	ISRAEL	7
USA	POLAND	7
USA	SWITZERLAND	7
ITALY	NETHERLANDS	6

© 2022. Guo W et al. Published online at http://www.aginganddisease.org/EN/10.14336/AD.2022.0620

ITALY	SWEDEN	6
UNITED KINGDOM	FINLAND	6
UNITED KINGDOM	NETHERLANDS	6
USA	INDIA	6
USA	KOREA	6
USA	MEXICO	6
USA	RUSSIA	6
USA	SPAIN	6
USA	SWEDEN	6
CANADA	SPAIN	5
CHINA	FRANCE	5
FRANCE	MEXICO	5
GERMANY	NETHERLANDS	5
ITALY	BELGIUM	5
ITALY	BRAZIL	5
ITALY	POLAND	5
ITALY	SPAIN	5
SPAIN	FRANCE	5
SPAIN	PORTUGAL	5

Supplementary Table 3. Keywords with a co-occurrence over 10.

Term	Occurrences	Relevance
older adult	1131	0.75
disease	982	0.23
sars cov	928	0.81
infection	664	0.65
participant	354	0.92
care	311	0.85
cell	300	1.18
loneliness	267	1.37
mechanism	239	0.61
virus	236	0.62
community	221	0.79
severity	209	0.41
experience	200	1.11
home	189	0.93
inflammation	189	0.99
program	176	0.77
survey	175	1.08
social isolation	167	1.06
technology	166	0.81
function	163	0.44
service	163	1.01
comorbidity	161	0.47
policy	159	0.91
cancer	143	0.24
mental health	140	0.99
expression	138	1.25
lockdown	137	1.21
score	136	0.74
crisis	134	0.81
anxiety	131	1.11
severe covid	131	0.79
access	127	0.89
physical activity	127	0.70
immune system	126	0.70
senescence	124	1.24

© 2022. Guo W et al. Published online at http://www.aginganddisease.org/EN/10.14336/AD.2022.0620

SUPPLEMENTARY DATA

ace2	123	1.28
pathway	123	0.77
severe acute respiratory syndrome coronavirus	122	0.72
immune response	121	0.98
complication	108	0.51
protein	106	1.21
obesity	105	0.55
depression	103	1.02
resource	103	0.72
scale	103	1.03
caregiver	101	1.26
t cell	99	1.21
practice	96	0.73
receptor	96	1.21
diabete	95	0.50
drug	95	0.78
family	93	0.63
vaccination	92	0.24
interaction	91	0.29
lung	91	1.27
world	91	0.46
hypertension	90	0.52
ageism	89	1.18
tissue	89	1.19
syndrome	86	0.53
dysfunction	84	0.87
gene	83	1.34
training	83	0.87
opportunity	82	0.60
recommendation	82	0.65
progression	81	0.73
damage	80	0.69
injury	80	0.66
questionnaire	80	1.11
viral infection	78	1.01
activation	77	1.17
place	77	0.83
immunity	76	0.82
susceptibility	74	0.49
china	72	0.64
immunosenescence	72	0.80
interview	72	1.15
social distancing	71	1.06
engagement	70	0.80
education	69	1.07
source	68	0.74
difficulty	67	1.20
isolation	67	0.64
contact	66	0.94
organization	66	0.35
regulation	66	0.31
variable	66	0.96
cytokine storm	65	1.03
pathogenesis	64	1.17
feeling	63	1.34
perception	63	1.17
pneumonia	63	0.55
respondent	62	1.29
feature	61	0.44
mouse	61	1.10
cellular senescence	60	1.32

© 2022. Guo W et al. Published online at http://www.aginganddisease.org/EN/10.14336/AD.2022.0620

infectious disease	60	0.46
marker	60	0.84
production	60	0.91
agent	57	0.83
attitude	57	1.12
clinical trial	57	0.34
resident	56	0.91
city	55	0.63
failure	55	0.30
provider	55	1.12
worker	55	0.82
distress	54	0.62
pathology	54	0.97
theme	54	1.26
elderly patient	53	0.54
manifestation	53	0.68
disease severity	52	0.91
restriction	52	0.74
body	51	0.33
cardiovascular disease	51	0.64
ratio	51	0.47
target	51	0.65
theory	51	0.64
telehealth	50	1.37
brain	49	1.04
guideline	49	0.72
melatonin	49	1.02
satisfaction	49	1.36
canada	48	0.95
concentration	48	0.88
enzyme	48	1.14
pathogen	48	0.80
alteration	47	0.78
facility	47	0.54
june	47	0.86
health care	46	0.76
human	46	0.45
implementation	46	0.76
phenotype	46	0.68
adherence	45	1.23
mobility	45	1.25
performance	45	0.84
combination	44	0.37
initiative	44	0.95
product	44	0.40
baseline	43	0.75
project	43	0.80
worry	43	1.37
fear	42	1.18
growth	42	0.41
hiv	42	0.82
molecule	42	1.20
researcher	42	0.75
younger adult	42	1.01
ageing	41	0.45
communication	41	0.74
demand	41	0.54
inequality	41	0.93
inflammaging	41	1.02
mask	41	2.17
metabolism	41	1.14
organ	41	0.80

© 2022. Guo W et al. Published online at http://www.aginganddisease.org/EN/10.14336/AD.2022.0620

SUPPLEMENTARY DATA

oxidative stress	41	1.14
stressor	41	0.72
staff	40	1.03
admission	39	0.61
depressive symptom	39	1.60
europe	39	0.92
inhibitor	39	1.28
sense	39	0.63
wave	39	0.78
government	38	0.78
homeostasis	38	0.99
adoption	37	1.00
brazil	37	0.85
cost	37	0.59
inflammatory response	37	1.07
involvement	37	0.47
member	37	0.98
participation	37	1.01
physician	37	0.64
potential	37	0.41
severe disease	37	0.55
strength	37	0.73
accumulation	36	1.16
host	35	1.02
parent	35	1.88
physical health	35	1.05
psychological distress	35	1.69
resistance	35	0.46
senescent cell	35	1.35
social support	35	1.36
acute respiratory distress syndrome	34	1.09
defense	34	0.85
friend	34	0.90
immune cell	34	1.06
institution	34	0.87
may	34	0.96
province	34	0.67
spike protein	34	1.52
web	34	0.61
chronic inflammation	33	0.99
economy	33	0.91
older patient	33	0.63
prognosis	33	0.71
systematic review	33	0.53
united state	33	1.11
animal	32	0.41
cytokine	32	1.21
habit	32	0.83
household	32	1.21
income	32	1.25
influenza	32	0.29
longitudinal study	32	0.93
lung disease	32	1.07
mirna	32	1.22
senior	32	1.23
ards	31	1.28
clinical outcome	31	0.79
cluster	31	0.72
dimension	31	0.62
dysregulation	31	0.78
fibrosis	31	1.38

© 2022. Guo W et al. Published online at http://www.aginganddisease.org/EN/10.14336/AD.2022.0620

first wave	31	1.10
pulmonary fibrosis	31	1.46
august	30	0.68
delivery	30	0.95
entry	30	0.68
family caregiver	30	1.44
fatality	30	0.37
macrophage	30	1.61
nursing home	30	0.74
provision	30	1.05
student	30	1.33
telemedicine	30	0.84
assistance	29	1.15
cognition	29	0.77
connection	29	0.78
disease progression	29	0.94
endothelial cell	29	1.40
quarantine	29	0.93
rna	29	1.04
speed	29	0.75
united states	29	0.97
vitamin d	29	0.72
compound	28	0.60
endothelial dysfunction	28	1.25
health system	28	1.37
october	28	0.95
odds ratio	28	0.55
quercetin	28	0.98
sarcopenia	28	0.40
significant difference	28	0.96
tea	28	1.53
consumption	27	0.47
copd	27	1.27
diabetes mellitus	27	0.40
intention	27	1.22
multimorbidity	27	0.87
session	27	1.35
supply	27	0.50
survival	27	0.79
wellbeing	27	1.42
fall	26	0.77
fever	26	0.27
lethality	26	0.87
odd	26	0.57
osteoporosis	26	0.47
pathophysiology	26	0.94
replication	26	1.32
user	26	1.04
utilization	26	1.19
volunteer	26	0.56
blood	25	1.12
help	25	1.19
logistic regression	25	0.60
metformin	25	1.13
online survey	25	1.39
respiratory disease	25	0.56
circumstance	24	0.76
cross sectional study	24	0.72
formation	24	0.76
immune function	24	0.81
pet	24	0.95
retirement	24	1.04

© 2022. Guo W et al. Published online at http://www.aginganddisease.org/EN/10.14336/AD.2022.0620

stroke	24	0.51
systemic inflammation	24	0.74
amount	23	0.66
control group	23	0.79
cov	23	0.88
gene expression	23	1.29
hallmark	23	0.80
hour	23	0.88
india	23	1.44
inflammatory cytokine	23	1.26
item	23	1.20
methodology	23	0.77
percentage	23	0.71
phenomenon	23	0.40
practitioner	23	0.94
race	23	0.90
discharge	22	0.64
dog	22	2.05
health care system	22	1.03
kidney	22	1.01
long term care	22	1.15
lymphocyte	22	0.93
middle	22	0.87
nroterve behavior	22	1.90
physical exercise	22	0.81
respiratory infection	22	0.51
senolytic	22	1.26
sleep	22	1.10
stereotype	22	1.32
angiotensin	21	1.22
animal model	21	0.80
apoptosis	21	1.27
confinement	21	0.98
data collection	21	1.32
delay	21	0.63
delay	21	0.63
estimate	21	0.56
expectation	21	1.55
face mask	21	3.09
family member	21	1.13
health service	21	1.28
healthcare	21	0.92
induction	21	1.32
integration	21	0.56
internet	21	1.28
medical care	21	1.72
mood	21	1.35
neurodegeneration	21	1.08
neurodegenerative disease	21	0.78
nurse	21	1.03
ontario	21	0.71
promotion	21	0.43
social contact	21	1.39
vulnerable population	21	0.85
beneficial effect	20	0.43
cell type	20	1.46
culture	20	0.60
dna	20	1.19
emotion	20	1.52
gerontology	20	0.91
host cell	20	1.19
inclusion	20	0.78

© 2022. Guo W et al. Published online at http://www.aginganddisease.org/EN/10.14336/AD.2022.0620

SUPPLEMENTARY DATA

molecular mechanism	20	0.99
neighborhood	20	1.52
neuron	20	1.24
new technoloav	20	1.36
proliferation	20	1.18
qol	20	1.43
racism	20	1.44
social connection	20	1.47
t cell response	20	1.28
telephone	20	1.09
acceptance	19	0.72
adaptive immune response	19	1.22
age difference	19	1.45
communication technology	19	1.18
daily life	19	1.07
demographic	19	0.71
future research	19	0.95
immune senescence	19	0.91
informal caregiver	19	1.81
japan	19	0.91
mean age	19	0.64
muscle	19	0.42
plasma	19	0.79
randomized controlled trial	19	0.71
rural area	19	0.86
sepsis	19	1.05
september	19	0.83
stakeholder	19	1.17
subjective age	19	1.30
sustainability	19	0.69
tmprss2	19	1.46
anti	18	1.01
chronic obstructive pulmonary disease	18	0.87
cns	18	1.11
cognitive	18	0.59
grandparent	18	4.66
heart	18	0.93
interleukin	18	1.08
long term care facility	18	0.81
mechanical ventilation	18	1.00
mitochondrial dysfunction	18	1.12
mortality risk	18	0.73
non covid	18	0.44
recruitment	18	0.58
relative	18	1.28
release	18	1.00
sasp	18	1.53
self perception	18	2.07
social determinant	18	1.27
variance	18	1.43
vulnerable group	18	1.04
anti inflammatory	17	1.12
autoimmunity	17	1.28
better understanding	17	0.57
black	17	1.77
brazilian longitudinal study	17	1.44

© 2022. Guo W et al. Published online at http://www.aginganddisease.org/EN/10.14336/AD.2022.0620

c reactive protein	17	0.68
case fatality rate	17	0.40
ckd	17	0.72
cognitive function	17	0.56
cough	17	0.33
cvd	17	0.62
cvd	17	0.62
expansion	17	0.37
feasibility	17	0.65
higher level	17	0.75
innate immune response	17	1.12
million	17	0.32
movement	17	0.74
nad	17	1.27
negative impact	17	1.42
nlr	17	2.12
protective measure	17	1.31
severe infection	17	0.74
singapore	17	1.20
telomere length	17	0.94
active aging	16	1.37
anxiety symptom	16	1.30
central nervous system	16	1.03
collaboration	16	1.44
complexity	16	0.81
elsi covd	16	1.59
employment	16	1.27
exacerbation	16	1.18
health behavior	16	0.84
iii	16	0.51
ipf	16	1.84
life space mobility	16	1.96
longevity	16	0.77
mci	16	1.14
mitochondrial function	16	1.20
mitochondrium	16	1.19
monocyte	16	1.17
physical function	16	1.02
policymaker	16	1.35
renin angiotensin system	16	1.42
research design	16	1.14
respiratory failure	16	1.15
sars cov2	16	0.79
semi	16	1.45
social medium	16	0.99
suicide	16	1.23
telephone interview	16	1.38
therapeutic target	16	1.35
usa	16	0.84
viral load	16	0.65
viral replication	16	1.22
vitro	16	1.34
ace	15	1.08
adjustment	15	0.82
asia	15	1.10
asthma	15	0.48
australia	15	0.82
belief	15	1.13
biological aging	15	0.35
building	15	0.59
cell death	15	1.22
decision making	15	0.83

© 2022. Guo W et al. Published online at http://www.aginganddisease.org/EN/10.14336/AD.2022.0620

dysbiosis	15	1.10
elder	15	0.94
elderly adult	15	0.38
first month	15	1.25
house	15	1.02
housing	15	2.07
human health	15	0.26
implications	15	0.90
major risk factor	15	0.69
microplastic	15	4.55
min day	15	1.34
national health	15	1.48
older worker	15	1.40
physical activity level	15	1.02
pilot study	15	0.89
population density	15	0.62
population density	15	0.62
severe case	15	0.95
spa	15	2.21
therapeutic	15	0.61
today	15	0.72
upregulation	15	1.32
adverse effect	14	0.28
autoimmune disease	14	0.87
average	14	0.92
cancellation	14	2.06
case study	14	1.02
co morbidity	14	0.58
confirmed case	14	1.05
hyperinflammation	14	1.28
innate immune system	14	1.24
intensive care unit	14	0.47
mitochondria	14	1.25
organism	14	0.73
potential mechanism	14	0.89
proposal	14	0.68
pwh	14	1.49
risk perception	14	1.61
spike	14	1.44
acceptability	13	1.49
aki	13	1.26
autonomy	13	1.34
caregiver burden	13	1.04
cognitive functioning	13	0.88
coronavirus infection	13	0.70
cross	13	0.92
degrees c	13	0.46
document	13	0.69
economic burden	13	1.63
elder abuse	13	1.62
ferritin	13	2.74
immunopathology	13	1.28
iran	13	0.68
key role	13	0.76
long term	13	1.38
ltss	13	1.80
medline	13	0.60
mrna	13	0.55
msc	13	1.49
negative effect	13	1.07
neurological manifestation	13	0.91

© 2022. Guo W et al. Published online at http://www.aginganddisease.org/EN/10.14336/AD.2022.0620

older age group	13	0.53
phq	13	1.52
physical distancing	13	1.44
primary outcome	13	0.76
rapamycin	13	1.23
respiratory viral infection	13	1.00
sars	13	0.55
scoping review	13	0.53
severe illness	13	0.50
shelter	13	1.21
start	13	0.82
successful aging	13	0.76
transportation	13	0.83
vaccine development	13	0.48
young adult	13	0.50
ace2 receptor	12	1.09
adaptive immunity	12	1.08
aged mouse	12	1.39
critical role	12	0.62
crp	12	0.83
current knowledge	12	0.60
dimer	12	1.05
dog walking	12	2.9
first time	12	0.59
further research	12	0.55
germany	12	1.14
globe	12	0.58
grandchild	12	5.33
gut microbiome	12	1.05
healthcare provider	12	1.28
heart failure	12	0.48
heating period	12	1.81
icu	12	0.50
infected patient	12	1.13
interferon	12	1.16
korea	12	2.08
later life	12	1.36
lockdown period	12	1.36
lower level	12	0.53
neurodegenerative disorder	12	0.91
pcrc	12	1.57
physical isolation	12	1.82
qualitative study	12	1.29
ras	12	1.34
recent study	12	0.91
rt pcr	12	0.48
secretory phenotype	12	1.55
severe acute respiratory syndrome	12	0.72
significant predictor	12	1.74
skin	12	1.02
social	12	1.66
social connectedness	12	1.35
suggestion	12	0.75
taiwan	12	0.53
usability	12	1.44
young person	12	0.64
younger patient	12	1.01
adipose tissue	11	1.07
case report	11	0.65
cfs	11	0.83

© 2022. Guo W et al. Published online at http://www.aginganddisease.org/EN/10.14336/AD.2022.0620

SUPPLEMENTARY DATA

chronic kidney disease	11	0.85
commentary	11	1.44
contagion	11	0.79
continuity	11	1.20
current study	11	0.54
disposable mask	11	4.24
elsi brazil	11	1.53
gut microbiota	11	1.11
hong kong	11	1.35
hospital mortality	11	1.05
hostile ageism	11	1.67
kind	11	1.09
mother	11	1.64
novel	11	0.84
online questionnaire	11	1.38
poor outcome	11	0.50
poor prognosis	11	0.96
race ethnicity	11	1.21
rationale	11	0.46
social interaction	11	1.24
urban area	11	1.12
canadian longitudinal study	10	1.48
current pandemic	10	0.61
dementia care	10	2.05
dendritic cell	10	1.32
everyday life	10	1.41
food insecurity	10	1.05
functional decline	10	0.70
healthy individual	10	0.61
median age	10	0.59
pathological condition	10	0.98
plwh	10	0.99
present review	10	0.53
social care	10	0.70

© 2022. Guo W et al. Published online at http://www.aginganddisease.org/EN/10.14336/AD.2022.0620
